# Supplementary figures and images for: Expression, not sequence, distinguishes miR-238 from its miR-239ab sister miRNAs in promoting longevity in Caenorhabditis elegans
Source: PLoS Genet. 2023 Nov 27;19(11):e1011055. doi: 10.1371/journal.pgen.1011055 (PMC10703411; doi:10.1371/journal.pgen.1011055)

Supplemental Figure 1. Predicted folding of wildtype and mutant miR-239ab primary transcripts

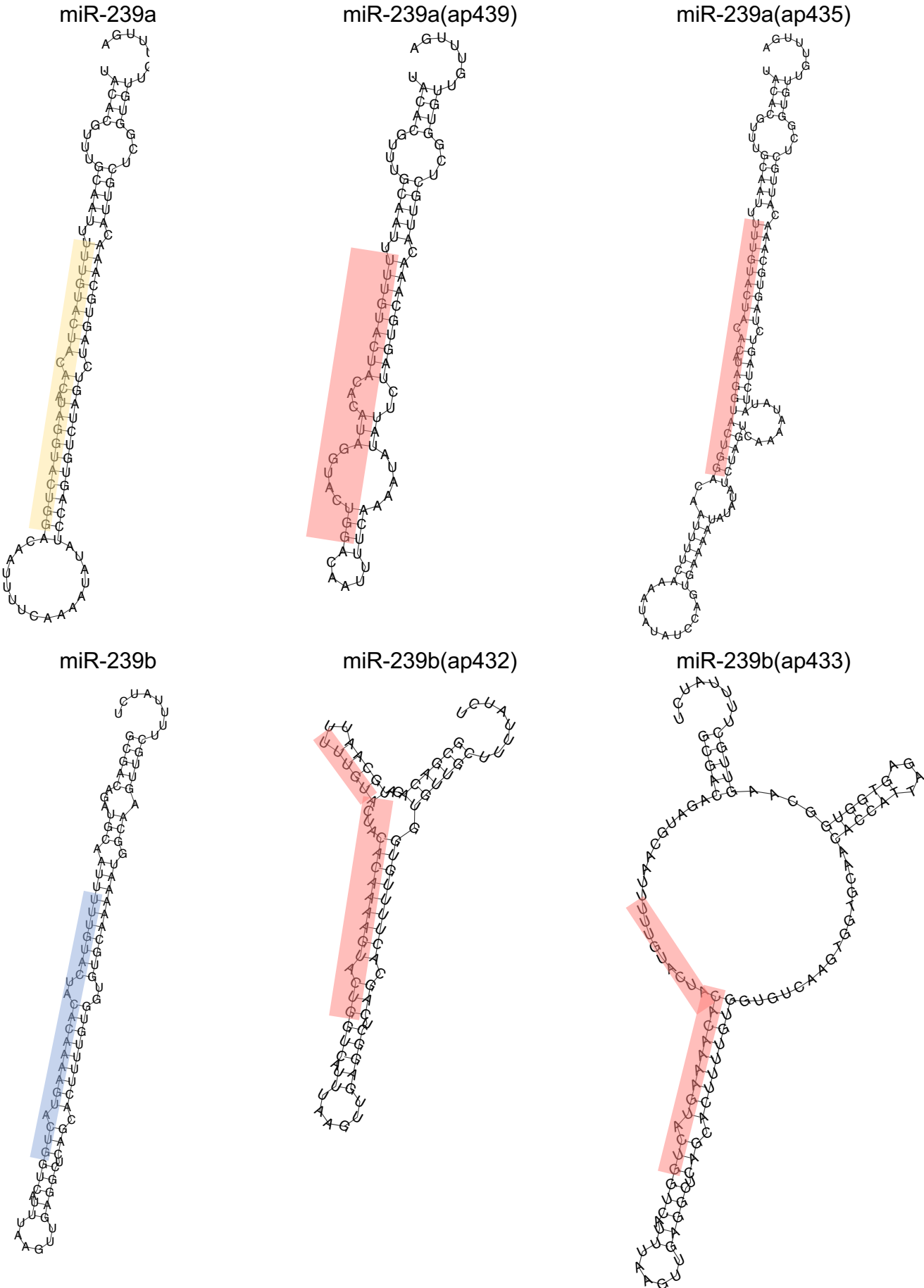

Supplement: S1 Fig — RNAfold structures for wildtype pre-miRNA structures of miR-239a and miR-239b have the mature miRNA sequence boxed in yellow and blue, respectively. New loss of function mutants generated in this study by CRISPR/Cas9 are indicated with mature miRNA sequences boxed in red. miR-239a(ap439) deletes 10 nucleotides in the 3’ arm of the stem. miR-239a(ap435) inserts 25 nucleotides into this region. miR-239b(ap432) deletes 15 nt at the base of the 3’ arm of the stem and miR-239b(ap433) deletes the GCAAAAA sequence and inserts 26 nt. (PDF) [file pgen.1011055.s006.pdf]
