# Supplementary material for: Expression, not sequence, distinguishes miR-238 from its miR-239ab sister miRNAs in promoting longevity in Caenorhabditis elegans
Source: PLoS Genet. 2023 Nov 27;19(11):e1011055. doi: 10.1371/journal.pgen.1011055 (PMC10703411; doi:10.1371/journal.pgen.1011055)

Supplemental Figure 2. Broodsize and heat shock survial for $_{p}$*miR-238::miR-239a* and $_{p}$*miR-238::miR-239b* strains.

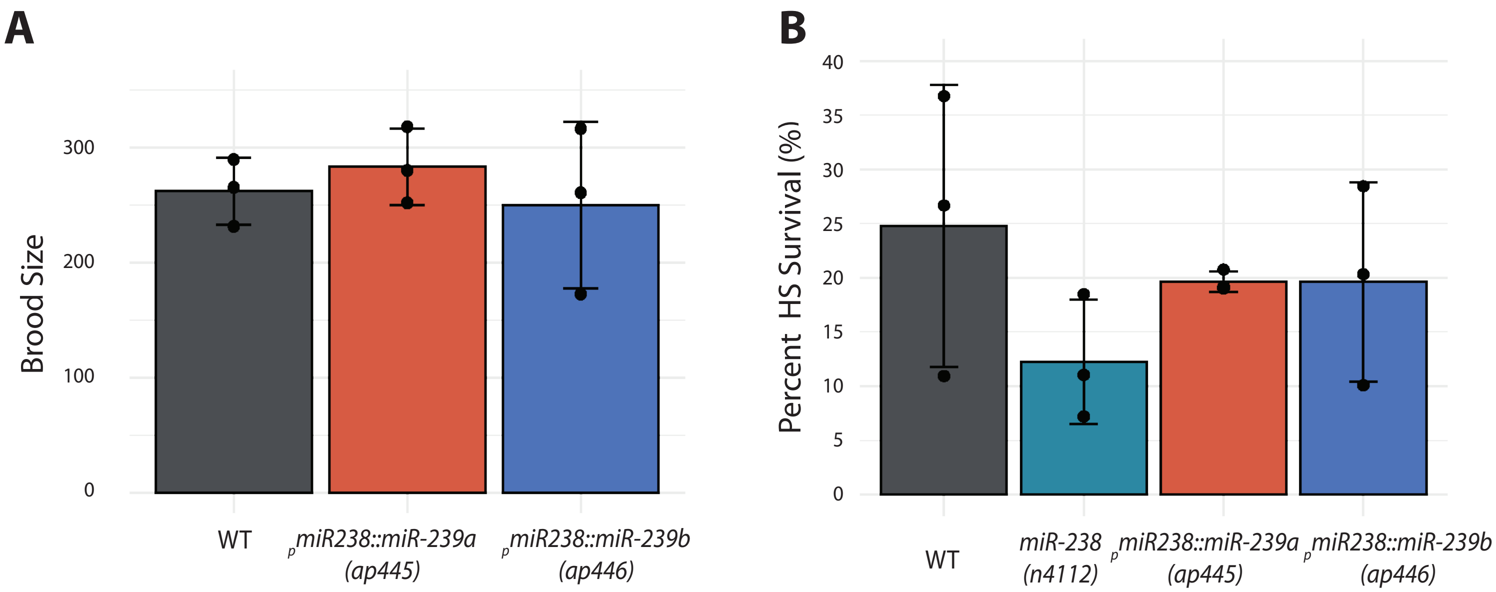

Supplement: S2 Fig — (A) Results from brood size analysis; miR-238(n4112) (aqua), pmiR-238::miR-239a (coral), and pmiR-238::miR-239b (blue) do not have a statistically significant difference when compared to WT (black). ANOVA and the post hoc test (Tukey’s HSD). Bar graph represents mean of three biological replicates; individual replicate data indicated with black dots. The error bars represent SDs. (B) Results from heat shock on day 2 adults for 15 hours at 32°C followed by recovery for 24hr at 20°C. miR-238 (n4112) (aqua), pmiR-238::miR-239a (coral), and pmiR-238::miR-239b (blue) do not have a statistically significant different percent heat shock survival when compared to WT (black). ANOVA and the post hoc test (Tukey’s HSD). Bar graph represents mean of three biological replicates; individual replicate data indicated with black dots. The error bars represent SDs. (PDF) [file pgen.1011055.s007.pdf]
